# Crystal structure of the *Arabidopsis* SPIRAL2 C-terminal domain reveals a p80-Katanin-like domain

**DOI:** 10.1371/journal.pone.0290024

**Published:** 2023-12-29

**Authors:** Derek L. Bolhuis, Ram Dixit, Kevin C. Slep

**Affiliations:** 1 Program in Molecular and Cellular Biophysics, University of North Carolina, Chapel Hill, North Carolina, United States of America; 2 Department of Biology and Center for Engineering Mechanobiology, Washington University in St. Louis, St. Louis, Missouri, United States of America; 3 Department of Biology, University of North Carolina, Chapel Hill, North Carolina, United States of America; University of Nova Gorica, SLOVENIA

## Abstract

Epidermal cells of dark-grown plant seedlings reorient their cortical microtubule arrays in response to blue light from a net lateral orientation to a net longitudinal orientation with respect to the long axis of cells. The molecular mechanism underlying this microtubule array reorientation involves katanin, a microtubule severing enzyme, and a plant-specific microtubule associated protein called SPIRAL2. Katanin preferentially severs longitudinal microtubules, generating seeds that amplify the longitudinal array. Upon severing, SPIRAL2 binds nascent microtubule minus ends and limits their dynamics, thereby stabilizing the longitudinal array while the lateral array undergoes net depolymerization. To date, no experimental structural information is available for SPIRAL2 to help inform its mechanism. To gain insight into SPIRAL2 structure and function, we determined a 1.8 Å resolution crystal structure of the *Arabidopsis thaliana* SPIRAL2 C-terminal domain. The domain is composed of seven core α-helices, arranged in an α-solenoid. Amino-acid sequence conservation maps primarily to one face of the domain involving helices α1, α3, α5, and an extended loop, the α6-α7 loop. The domain fold is similar to, yet structurally distinct from the C-terminal domain of Ge-1 (an mRNA decapping complex factor involved in P-body localization) and, surprisingly, the C-terminal domain of the katanin p80 regulatory subunit. The katanin p80 C-terminal domain heterodimerizes with the MIT domain of the katanin p60 catalytic subunit, and in metazoans, binds the microtubule minus-end factors CAMSAP3 and ASPM. Structural analysis predicts that SPIRAL2 does not engage katanin p60 in a mode homologous to katanin p80. The SPIRAL2 structure highlights an interesting evolutionary convergence of domain architecture and microtubule minus-end localization between SPIRAL2 and katanin complexes, and establishes a foundation upon which structure-function analysis can be conducted to elucidate the role of this domain in the regulation of plant microtubule arrays.

## Introduction

Microtubules are polarized cytoskeletal polymers of the αβ-tubulin heterodimer that undergo dynamic instability [1, 2]. Microtubules are critical for cellular support and the asymmetric localization of cellular factors either through polarized microtubule motor-dependent transport, or via factors that specifically bind the microtubule plus or minus end. Collectively, asymmetric functions are best achieved when microtubules are arranged in an array that can adapt and reorient in response to intrinsic (e.g. cell cycle regulators) or extrinsic (e.g. a chemoattractant) cues. While some organisms use centrosomes to organize microtubule arrays, many organisms and cell types form acentrosomal microtubule arrays. How these arrays form, are maintained over time, and morph or reorient in response to cues is poorly understood. Higher plants form acentrosomal cortical interphase microtubule arrays that aid in the asymmetric localization of cell wall biosynthesis machinery, a process critical for anisotropic growth and development [3–6]. In many tissues, plant acentrosomal microtubule arrays respond to cues including light. For example, perception of blue light by hypocotyl epidermal cells leads to reorganization of the microtubule array from a net lateral orientation to a net longitudinal orientation as part of the photomorphogenesis pathway.

Plant cortical microtubule array reorganization requires a set of microtubule regulatory proteins. Along the initial lateral microtubule array, γ-tubulin complexes nucleate new microtubules oriented at an angle from the parental microtubule. Additional γ-tubulin complexes bind these nascent microtubules, leading to the nucleation and polymerization of a set of microtubules arranged orthogonal to the parental lateral array. The orthogonal positioning of microtubules yields microtubule intersections termed crossover sites. The microtubule severing enzyme, katanin, is recruited to nucleation and crossover sites, where the nascent/longitudinally-oriented microtubule is severed, and its minus end stabilized by the protein SPIRAL2 (SPR2) [7–11]. The preferential severing and minus-end stabilization of longitudinal microtubules leads to their polymerization and amplification over the parental lateral array. How plant cytoskeletal regulators recognize microtubule minus ends and crossover sites and differentiate lateral versus longitudinal microtubules is poorly understood.

**Fig 1:**
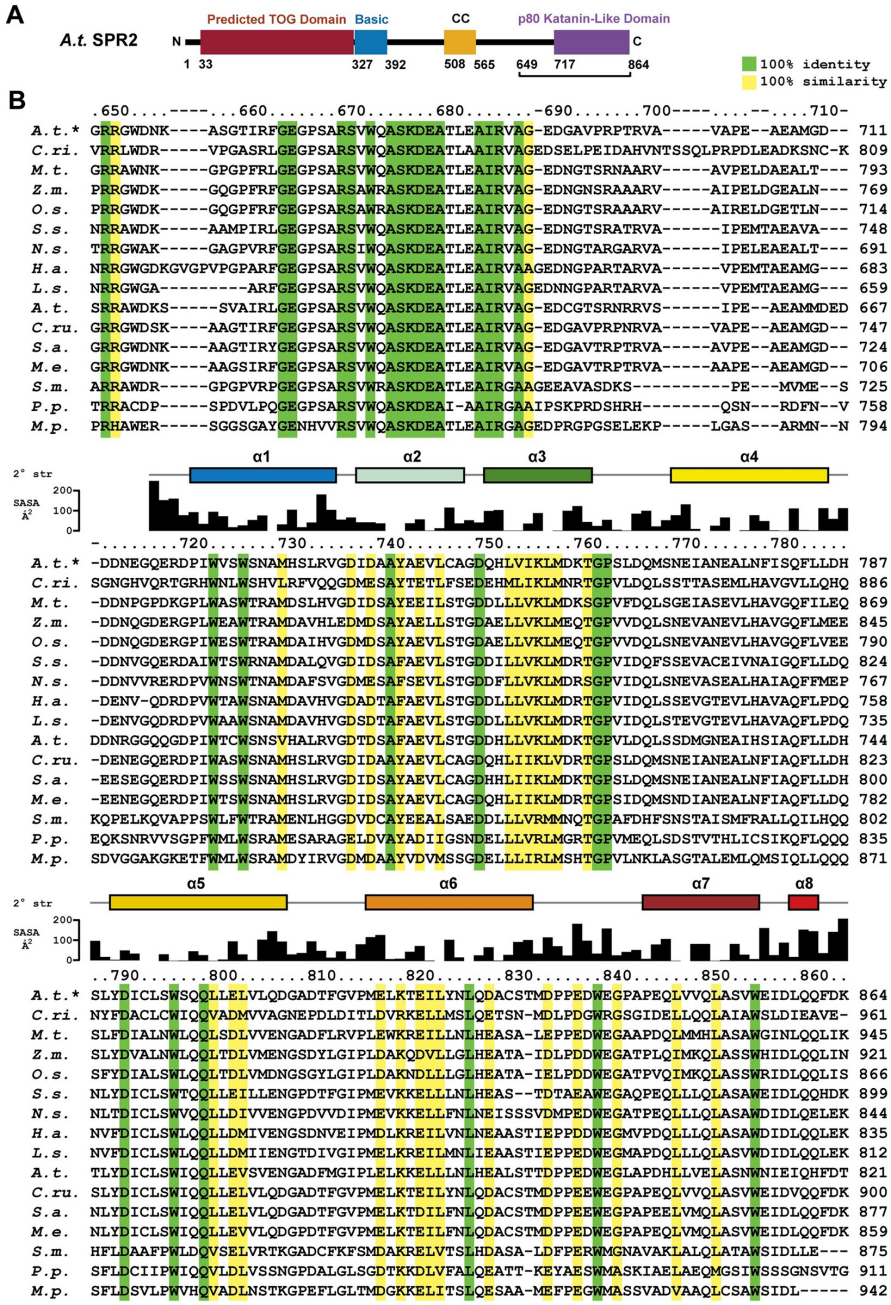
SPR2 contains a conserved C-terminal domain. (A) Domain architecture of SPR2, consisting of a predicted N-terminal TOG domain, a basic region, a central coiled coil, and a conserved C-terminal domain that structurally resembles the p80 katanin domain involved in p60-p80 katanin heterodimerization. The construct used for crystallization (residues 649–864) is indicated by a bracket. (B) Sequence alignment of SPR2 homologs from diverse land plants. Conservation is mapped on the sequence alignment as follows: green, 100% identity across species aligned; yellow, 100% similarity across species aligned using the following similarity rubric (LIVM, TSC, RK, NQ, DE, FYW, AG, H, P). Residue numbers are indicated above the alignment for *A*.*t*. SPR2, as are secondary structure and residue solvent accessibility, both determined based on the crystal structure of the *A*.*t*. SPR2 C-terminal domain presented here. Aligned species: *Arabidopsis thaliana* (*A*.*t*., thale cress), *Ceratopteris richardii* (*C*.*ri*., triangle waterfern), *Musa troglodytarum* (*M*.*t*., fe’i banana), *Zea mays* (*Z*.*m*., maize), *Oryza sativa* (*O*.*s*., Asian rice), *Spatholobus suberectus* (*S*.*s*., millettia vine), *Nicotiana sylvestris*, (*N*.*s*., flowering tobacco), *Helianthus annuus* (*H*.*a*., sunflower), *Lactuca sativa* (*L*.*s*., butterhead), *Capsella rubella*, (*C*.*ru*., pink shepherd’s-purse), *Sinapis alba* (*S*.*a*., white mustard), *Microthlaspi erraticum* (*M*.*e*., erratic small pennycress), *Selaginella moellendorffii* (*S*.*m*., spikemoss), *Physcomitrium patens* (*P*.*p*., spreading earthmoss), and *Marchantia polymorpha* (*M*.*p*., umbrella liverwort). The SPR2 sequence is presented at the top of the alignment (denoted: *A*.*t*.*), while the sequence of the *A*.*t*. SPR2-like protein (SP2L) is presented in the middle of the alignment.

SPR2 (also known as TORTIFOLIA1 and CONVOLUTA) was identified as a factor involved in anisotropic growth in *Arabidopsis thaliana* (*A*.*t*.), with mutations leading to right-handed spiral growth [12]. Initial investigations demonstrated that SPR2 colocalizes with cortical microtubules, has in vitro microtubule binding activity, affects microtubule dynamics and microtubule array reorientation, and modulates microtubule severing [13–16]. Subsequent investigations found that SPR2 family members bind and stabilize the microtubule minus end, both in vivo and when examined using in vitro microtubule dynamics reconstitution assays [9–11]. In metazoans, CAMSAP protein family members bind and regulate microtubule minus ends using a CKK domain [17–21]. Higher plants lack CAMSAP proteins, but have members of the plant-specific SPR2 family [22]. The domain architecture of SPR2 family members is distinct from CAMSAP proteins, as the former contains a predicted N-terminal TOG domain [23–26], a central coiled-coil, and a helical C-terminal domain of unknown structure (Fig 1A). The structure and mechanism of SPR2 microtubule minus end recognition and regulation is a central question in plant cytoskeletal research which requires structural and functional analysis of each conserved domain. Two large linker regions (~100 residues each) flank the central coiled coil domain. As the sequences of these two linker regions are not conserved across species, we hypothesize that they serve a role to distally tether the conserved domains to one another. As large disordered linkers are likely to hinder crystallization of the full-length protein, we set out to determine the structure of a single, conserved SPR2 region: the SPR2 C-terminal domain. Two *SPR2* alleles with a right-handed twisting growth phenotype: *spr2-4*, and *tor1-10*, have T-DNA insertions that cause frameshifts at SPR2 residues 627 and 630 respectively [13, 14]. These insertions compromise proper translation of the SPR2 C-terminal region (residues 649–864), highlighting the importance of the C-terminal region in SPR2 function.

Here, we explore the structure of the SPR2 C-terminal domain using x-ray crystallography. The aim of the study was to determine the oligomeric state and structure of the SPR2 C-terminal conserved region, compare and contrast the structure with other microtubule associated protein domains, and map conservation on the domain to identify tentative protein-protein interaction sites. We find that the *A*.*t*. SPR2 C-terminal domain is monomeric in solution, and we present the 1.8 Å resolution crystal structure of the domain, which reveals an α-solenoid fold consisting of seven conserved α-helices. Comparison of the SPR2 C-terminal domain structure with similar domain folds from Ge-1 and katanin p80 highlights distinct topological features of SPR2 indicative of distinct function. We identify a conserved face of the SPR2 C-terminal domain likely involved in binding protein partners.

## Materials and methods

### Sequence alignment

SPR2 and homologs from diverse land plant species were aligned using the Clustal Omega server [27]. The resulting alignment was adjusted manually using the SPR2 structure as a guide for conserved secondary structure elements. Secondary structure prediction used the Jpred4 server [28].

### Protein expression and purification

*A*.*t*. SPR2 DNA encoding residues 649–864 was generated using the polymerase chain reaction method (primers: 5’-GGCAGGACCCATATGGGCAGGAGAGGGTGGGATAATAAAGC-3’ and 5’-GCCGAGCCTGAATTCTTACTTGTCGAACTGTTGGAGATCGATTTC-3’) and individually sub-cloned into pET28 (Millipore Sigma, Burlington, MA) using engineered NdeI and EcoRI restriction endonuclease sites, digested, and ligated (New England Biolabs, Ipswich, MA). The construct was transformed into B834(DE3) *E*. *coli* methionine auxotrophic cells, grown to an optical density at 600 nm of 1.0 in 6l SelenoMet Medium (Molecular Dimensions Limited, Rotherham, UK) containing 50 μg/l kanamycin, 100 μM iron sulfate, and 60 mg/l DL-selenomethionine (Millipore Sigma), the temperature lowered to 20° C, and protein expression induced with 100 μM Isopropyl β-D-1-thiogalactopyranoside for 12 hours. Cells were harvested by centrifugation, and resuspended in 150 ml buffer A (25 mM Tris pH 8.0, 300 mM NaCl, 10 mM imidazole, 0.1% (v/v) β-mercaptoethanol, 5 mM L-methionine) at 4° C, supplemented with DNase (5 μg/ml final concentration, Worthington Biochemical Corp., Lakewood, NJ), lysozyme (10 μg/ml final concentration, Thermo Fisher Scientific, Waltham, MA), and 0.5 mM phenylmethylsulfonyl fluoride (PMSF). Lysis was aided by sonication during which the PMSF final concentration was increased to 1 mM. Lysate was cleared by centrifugation at 23,000 x g for 45 minutes at 4° C. The supernatant was loaded onto a Ni^2+^-NTA column (QIAGEN, Hilden, Germany) and washed with 750 mls of buffer A. Protein was batch eluted with buffer B (buffer A supplemented with 290 mM Imidazole). CaCl_2_ was added to 1 mM final concentration, and 0.1 mg bovine α-thrombin (Haematologic Technologies, Essex Junction, VT) added to proteolytically cleave off the N-terminal His_6_ tag, leaving an N-terminal Gly-Ser-His-Met N-terminal cloning artifact. Protein was dialyzed into buffer A for 24 hrs using 3k MWCO dialysis tubing (Thermo Fisher). Protein was then filtered over a benzamidine-Sepharose column (Cytiva, Marlborough, MA) to remove thrombin. A subsequent Ni^2+^-NTA column was used to remove uncleaved His_6_-tagged protein. Cleaved protein was buffer exchanged into storage buffer (25 mM Tris pH 8.5, 500 mM NaCl, and 0.1% β-mercaptoethanol, 5 mM L-methionine), concentrated using 3 kDa Amicon Ultra Spin Concentrators (MilliporeSigma) to 2.8 mM (68 mg/ml), flash frozen in liquid nitrogen, and stored at -80° C.

### Size exclusion chromatography and multi-angle light scattering

The SPR2 649–864 construct (100 μl of 220 μM protein) was injected onto a Superdex 200 10/300 GL size exclusion column (Cytiva) pre-equilibrated and run in 25 mM Tris pH 8.5, 500 mM NaCl, 0.1% β-mercaptoethanol, 0.2 g/L sodium azide. The protein sample was then directly passed through a Wyatt DAWN HELEOS II light scattering instrument and a Wyatt Optilab rEX refractometer. The light scattering values and the refractive index values were used to calculate the weight-averaged molar mass (M_W_) across the elution peak using the Wyatt Astra V software program (Wyatt Technology Corp., Santa Barbara, CA). Data plots were generated using Prism (GraphPad Software, San Diego, CA). Data shown are representative of duplicate runs.

### Protein gel analysis

Purified SPR2 C-terminal region protein (native and SeMet-substituted, load: 5 μg/well each), as well as SeMet-substituted SPR2 C-terminal region crystals were analyzed using sodium dodecyl sulfate (SDS) polyacrylamide gel electrophoresis (PAGE) on a 15% polyacrylamide gel followed by Coomassie blue staining. For the analysis of crystals, 15 SRP2 C-terminal region SeMet-substituted crystals (grown contemporaneously with the crystal used for diffraction data collection) were individually harvested, washed three times in well solution (1.05 M Ammonium sulfate, 100 mM sodium acetate pH 4.6), dissolved in SDS gel loading buffer, heated (5 min., 95° C), and loaded into a well.

### Protein crystallization

Selenomethionine (SeMet)-substituted SPR2 (residues 649–864) was crystallized using the hanging drop procedure at 20° C. 2 μl of SPR2 protein at 7 mg/ml was mixed with 2 μl of well solution (1.05 M Ammonium sulfate, 100 mM sodium acetate pH 4.6), placed on a silanized glass coverslip, and used to seal a chamber containing 1 ml of the well solution. Crystals formed overnight and continued to grow over the course of a week. Single crystals were harvested, transferred to FOMBLIN Y (MilliporeSigma), flash frozen in liquid nitrogen, and stored in liquid nitrogen.

### Data collection, structure determination, refinement, and analysis

A selenium SAD peak data set at 12,661.01 eV (λ = 0.9792603 Å) was collected on a single crystal to a resolution of 1.8 Å. Diffraction data were collected at the Advanced Photon Source beamline 22-ID at 100 K in 0.5° oscillations, across 360°. Crystals belong to the P2_1_2_1_2_1_ space group with one molecule in the asymmetric unit. Data were indexed, integrated, and scaled using HKL2000 [29]. Selenium sites were identified and used to generate initial density-modified electron density maps using PHENIX AutoSol [30]. Initial models were built using AutoBuild (PHENIX), followed by reiterative manual building in Coot and refinement using phenix.refine [30, 31]. The SeMet-substituted structure was refined against an MLHL target function. The free *R* used 10% of the data randomly excluded from refinement. Information regarding data statistics, model building, and refinement is presented in Table 1. Electrostatics was calculated using APBS [32]. Protein Data Bank (PDB) structure similarity searches were performed using the Dali server [33]. Pairwise structural alignments and rmsd values were calculated using the PDBeFold server [34]. Solvent accessibility was calculated using the PDBePISA server [35]. Structure figures were generated using PyMOL (Schrödinger, New York, NY).

**Table 1:** Crystallographic data processing and refinement statistics.

Crystal	*A*.*t*. SPR2 residues 649–864
**Data Collection**	
Wavelength (Å)	0.9792603
Space group	P 2_1_ 2_1_ 2_1_
Cell dimensions: a, b, c (Å)	35.5, 47.7, 111.1
Cell dimeinsions: α, β, γ (°)	90, 90, 90
Resolution (Å)	50.00–1.80 (1.86–1.80)
# Reflections: Measured / Unique	187,724 (10,242) / 18,011 (1679)
Completeness (%)	98.3 (92.9)
Mean redundancy	10.4 (6.1)
<I/σI> (Xtriage)	14.2 (1.9)
R_sym_	0.093 (0.241)
R_meas_	0.098 (0.263)
R_pim_	0.030 (0.102)
CC1/2	0.994 (0.973)
CC*	0.998 (0.993)
**Refinement**	
Resolution (Å)	33.80–1.80 (1.85–1.80)
R/ R_free_ (%)	18.6 (20.9) / 21.7 (28.2)
# Reflections, R/R_free_	16121 (1113) / 1792 (123)
Total atoms: Protein / Water	1173 / 95
Wilson B factor (Å^2^)	22.9
Average B factors: all / protein atoms / waters	28.2 / 27.3 / 39.4
F_o_,F_c_ correlation	0.95
Residues modeled	717–864
Stereochemical ideality (rmsd): Bonds (Å) / Angles (°)	0.011 / 1.323
Ramachandran Analysis: Favored / Allowed (%)	99.3 / 0.7
PDB Accession Code	8F8N

Values in parentheses indicate statistics for the highest-resolution shell.

## Results and discussion

### The SPR2 C-terminal region is highly conserved across land plants

To gain insight into the structure of the SPR2 C-terminal region, we aligned SPR2 homologs from diverse land plants including bryophytes such as liverwort (*M*.*p*.) and spreading earthmoss (*P*.*p*.), and vascular plants such as spikemoss (*S*.*m*.), Asian rice (*O*.*s*.), and thale cress (*A*.*t*.) (Fig 1B). SPR2 homologs aligned well over this C-terminal region, with a cluster of sequence identity corresponding to *A*.*t*. SPR2 residues 664–689, and across the region spanning 723–855. A segment of low identity and variable length bridges these two regions across the species aligned. Overall, across the ≥450 million years of divergence represented by these species [36, 37], their SPR2 homologs have about 13% sequence identity across the C-terminal region. Based on this conservation, we cloned a SPR2 construct embodying residues 649–864, expressed the construct in *E*. *coli*, and purified the protein to homogeneity.

**Fig 2:**
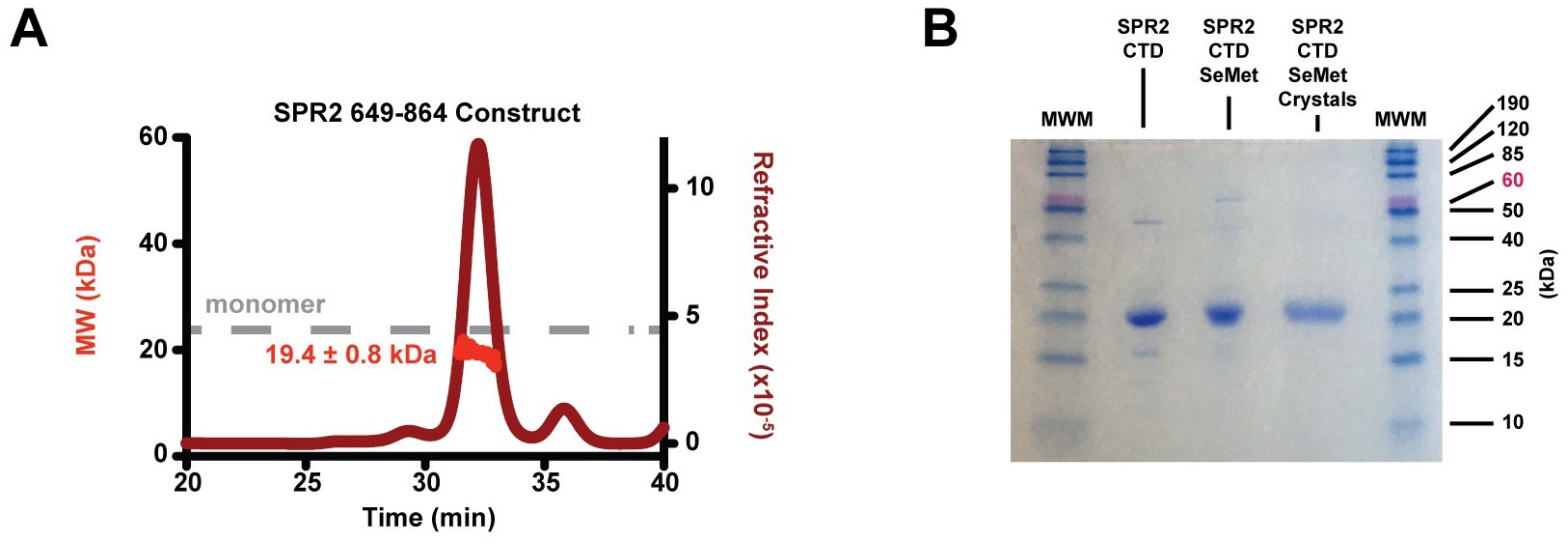
The SPR2 C-terminal domain is monomeric. A) SECMALS analysis of the purified SPR2 649–864 construct (full-length FW: 24.2 kDa). Plot shows the elution profile from the size exclusion column as measured using the refractive index (y-axis at right) over time. The experimentally determined mass is plotted in kDa (MW, y-axis at left) over time across the elution peak. The average mass (± standard deviation) is indicated. The dashed gray line indicates the monomeric formula weight of a full-length construct (24.2 kDa). B) Coomassie-stained SDS PAGE (15%) analysis of purified native (not SeMet-substituted) and SeMet-substituted SPR2 C-terminal domain constructs, and harvested SeMet-substituted SPR2 crystals. Molecular weight marker (MWM) standards are indicated.

To determine whether the SPR2 C-terminal region is monomeric or oligomeric, we analyzed the construct using size exclusion chromatography multi angle light scattering (SECMALS) (Fig 2A). The SPR2 C-terminal region eluted as one main peak with an experimentally determined mass of 19.4 ± 0.8 kDa. The SPR2 649–864 construct has a formula weight of 24.2 kDa. Thus, the SECMALS-determined mass of 19.4 kDa indicates that the SPR2 C-terminal region construct is monomeric at the concentration examined, but may be degraded. To further investigate the possibility of degradation, we analyzed the purified SPR2 C-terminal region protein using SDS PAGE (Fig 2B). The SPR2 C-terminal region protein band migrated at ~20 kDa, aligned with the SECMALS experimentally determined mass, and suggestive of degradation, potentially due to a cryptic thrombin protease site that was cleaved during thrombin-treatment.

### The SPR2 C-terminal domain is a conserved, 7-helix α-solenoid

**Fig 3:**
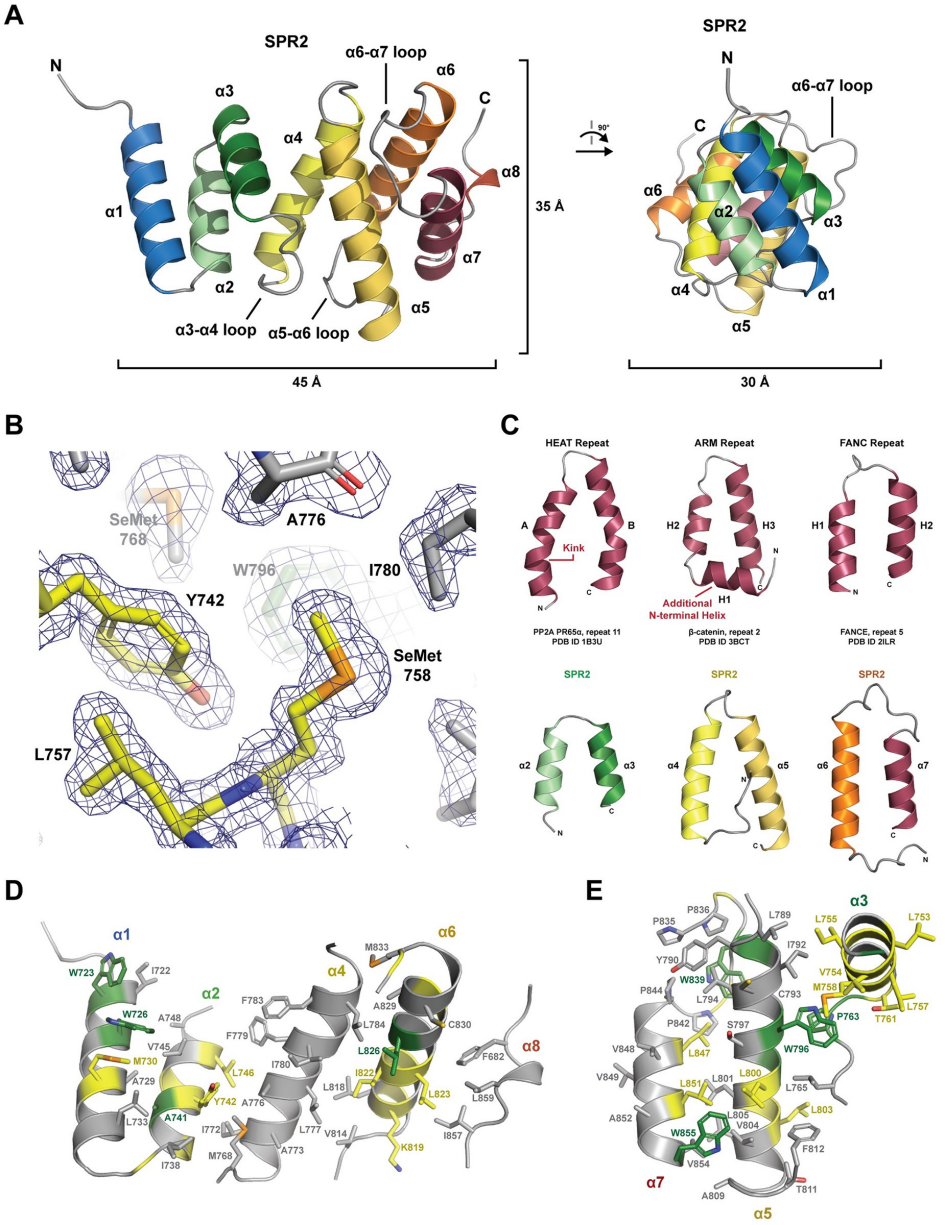
The SPR2 C-terminal domain is an α-solenoid helix-turn-helix domain containing seven helices. (A) Structure of the *A*.*t*. SPR2 C-terminal domain shown in cartoon format. The seven core helices of the domain (α1-α7) are colored across the spectrum. A final helix, α8, packs against the domain, but is not conserved across SPR2 homologs, and thus is not considered part of the core domain. Six of the helices form helix-turn-helix pairs: 2α-3α, 4α-5α, and 6α-7α. View at left is rotated 90° about the y-axis to generate the view at right. Relative dimensions of the domain are indicated. (B) Conserved residues in the α2-α3 region contribute the domain’s hydrophobic core. Residues are shown in stick format, with conservation colored as in Fig 1B, with final 2mF_o_-DF_c_ electron density shown in blue, contoured at 1.0 σ. Two SeMet residues used in phasing are indicated. (C) Structural comparison of canonical ARM, HEAT, and FANC repeats (top row) versus SPR2 C-terminal domain helix-turn-helix pairs α2-α3, α4-α5, and α6-α7 (bottom row). The representative HEAT repeat is from the structure of human PP2A PR65α (PDB ID 1B3U), repeat number 11 [38], with the canonical kink in helix A delineated. The representative ARM repeat is from the structure of mouse β-catenin (PDB ID 3BCT), repeat number 2 [39], with the canonical additional N-terminal helix H1 delineated. The representative FANC repeat is from the structure of human FANCE (PDB ID 2ILR), repeat number 5 [40]. (D-E) Splayed view of the SPR2 C-terminal domain, showing residues (primarily hydrophobic) buried in the core, contributed from helices α1, α2, α4, α6, and α8 (D), and helices α3, α5, and α7 (E), which respectively constitute opposite regions of the domain. The side chains of core residues are shown in stick format, colored based on the conservation delineated in Fig 1B.

To gain insight into the architecture of the SPR2 C-terminal region, we crystallized the *A*.*t*. SPR2 649–864 construct and determined its three-dimensional structure. We expressed, purified, and crystallized SeMet-substituted SPR2 649–864. Similar to the native protein, SDS PAGE analysis of purified and crystallized SeMet-substituted protein yielded respective bands that each migrated at ~20 kDa (Fig 2B). We collected a single wavelength anomalous diffraction (SAD) data set at the selenium peak to 1.8 Å resolution. The crystal belonged to the space group P2_1_2_1_2_1_, with one SPR2 molecule in the asymmetric unit, and a solvent content of 35% (calculated using the complete SPR2 649–864 construct, thus representing a lower limit for the solvent content if the construct was degraded) (Fig 3). Selenium sites were identified and used to phase the structure, yielding clear, interpretable electron density (Final 2mF_o_-DF_c_ electron density shown in Fig 3B), for which residues 717–849 (which includes the construct’s carboxy-terminal residue) were modeled. No electron density was apparent for the region N-terminal to residue 717. The absence of electron density for the 649–716 region may be due to intrinsic disorder and/or N-terminal proteolytic cleavage or degradation. As SECMALS and SDS PAGE analysis revealed a purified and crystallized protein of ~20 kDa (Fig 2), and the crystal structure includes the construct’s C-terminal residue, we predict that thrombin treatment resulted in cleavage at a cryptic site in the N-terminal region (potentially after R686 or R696). This means that the highly conserved region spanning residues 664–689 is mostly, or completely removed from the purified protein. The C-terminal region of the SPR2 construct modeled accounts for 16.7 kDa, indicating that an N-terminal segment of ~3 kDa is present, but disordered in the crystal lattice. The final model was refined to an R value of 18.6%, and a R_free_ value of 21.7%. See Table 1 for crystallographic and refinement statistics.

SPR2 residues 717–849 form a right-handed α-solenoid helix-turn-helix structure, composed of seven conserved α-helices (α1-α7) (Fig 3A). The dimensions of the domain are approximately 45 Å along the axis of the solenoid, 35 Å high, and 30 Å wide. A short helix, α8, packs against the domain, but this segment is not conserved across SPR2 homologs (Fig 1B) and is thus not considered part of the domain’s core structure. Six of the seven α-helices form anti-parallel helix-turn-helix pairs (α2-α3, α4-α5, and α6-α7) that pack against one another. A number of helix-turn-helix motifs form α-solenoid structures including Huntingtin, Elongation factor 3, protein phosphatase 2A, TOR1 (HEAT), armadillo (ARM), and FANC repeats [40] (Fig 3C). Of these repeats, the SPR2 C-terminal domain helix-turn-helix motifs are structurally most similar to FANC repeats, as the helices are relatively straight, and lack a canonical kink present in the first helix (helix A) of a HEAT repeat, or the additional N-terminal helix (helix H1) of an ARM repeat. The kink in the HEAT repeat structure is due to a proline residue in the first helix, while the separate N-terminal helix of ARM repeats is delineated by a position specific glycine and proline residue that position the N-terminal helix orthogonal to the axes of the subsequent two helices. The helix-turn-helix motifs of the SPR2 C-terminal domain lack these specific proline and glycine residues. The two helices in each pair form a hydrophobic interface between each other, and with the flanking helices, collectively form a hydrophobic core that runs along the axis of the α-solenoid (Fig 3B, 3D and 3E). The loops between helices vary in length, both within a helix-turn-helix motif, and between these motifs. Extended ordered loops of conserved length include the α3-α4 loop, the α5-α6 loop, and the α6-α7 loop (Fig 3A).

**Fig 4:**
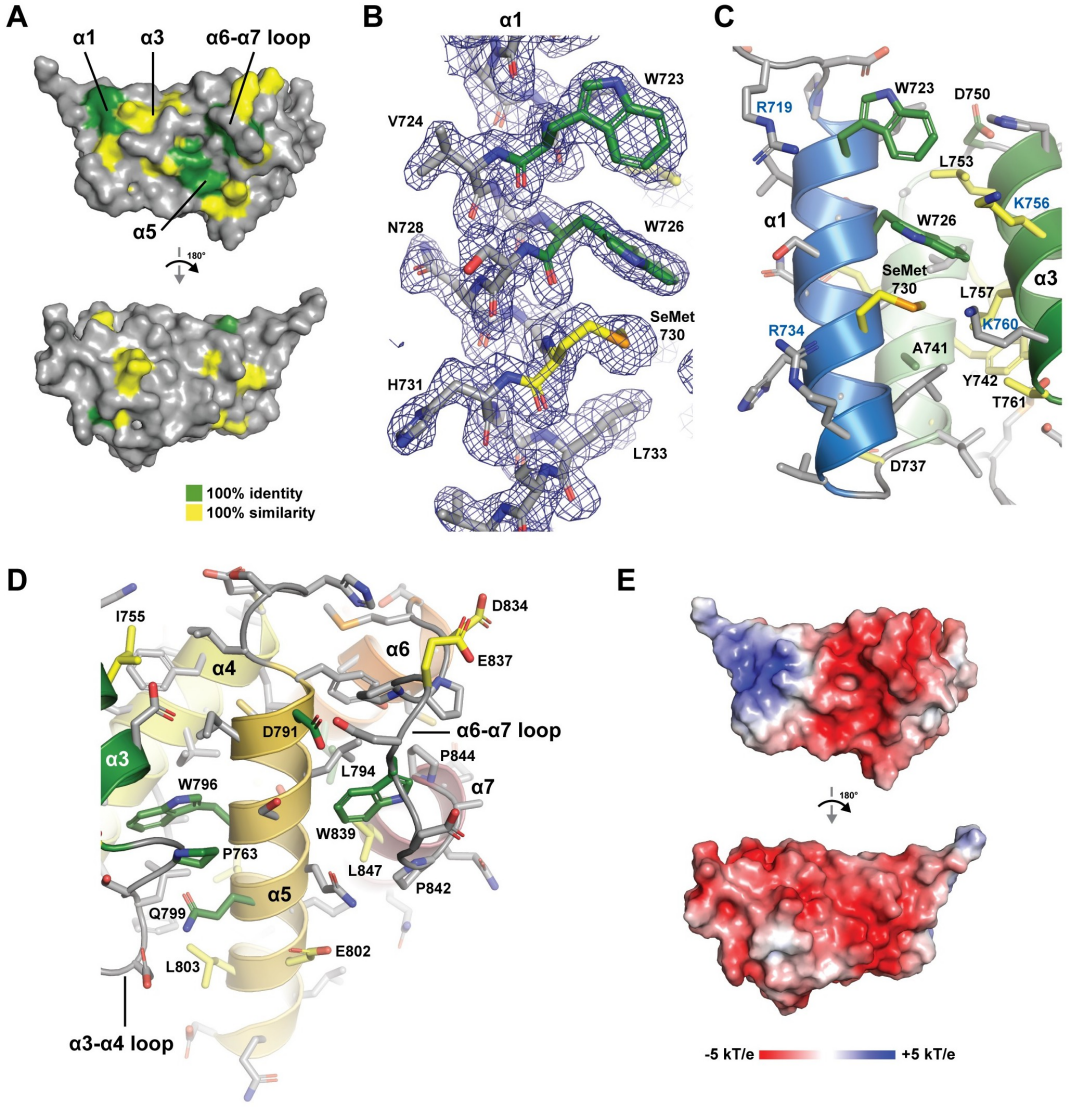
The SPR2 C-terminal domain has a conserved face with partitioned charge. (A) The SPR2 C-terminal domain shown in surface representation, with conservation from Fig 1B mapped on the surface (green: 100% identity; yellow: 100% similarity). Orientation at top as shown in Fig 3A (image at left), orientation below after a 180° rotation about the y-axis. (B) Conserved, hydrophobic, surface exposed determinants of the α1 helix (W723, W726, and SeMet730) are shown in stick format. Conservation is colored as in Fig 1B, with final 2mF_o_-DF_c_ electron density shown in blue, contoured at 1.0 σ. (C) View of conserved residues in the α1–α3 region, highlighting the surface exposed hydrophobic and basic nature of the region. The basic residues R719 and R734 of *α*1, and K756 and K760 of *α*3 are labeled in blue font. Backbone shown in cartoon format, colored as in Fig 3A, with residues shown in stick format, colored as in Fig 1B. (D) View of conserved residues in the α3–α7 region, highlighting the conserved, surface exposed residues along α5, residue P763 of the α3-α4 loop, as well as residue W839 of the α6-α7 loop, which is positioned in a pocket on the side of the domain. The W839 side chain interacts with and is stabilized by residues that include L794, P842, P844, and L847. Backbone shown in cartoon format, colored as in Fig 3A, with conserved residues shown in stick format, colored as in Fig 1B. (E) Electrostatic surface potential mapped on the SPR2 C-terminal domain structure, views oriented as in A.

Conservation, as contoured in Fig 1B, maps primarily to one face of the domain, with a high degree of identity conserved over ≥450 million years of evolution (Fig 4A). Key contributions to this conserved face come from surface-exposed hydrophobic residues on α1 (Fig 4B and 4C), including W723, W726, M730, and a cluster of hydrophobic residues on the α3-α4 loop—α5 interface, including L803 and P763, which stacks against W796 (Fig 4D). The α6-α7 loop forms an extensive projection from the domain that packs against α5, forming a hydrophobic pocket involving W839 (Fig 4D). W839 is stabilized by a number of surrounding hydrophobic residues, including L794, P842, P844, and L847 (Fig 4D). The domain has a net negative charge (Fig 4E). On the conserved face of the domain, charge is partitioned, with a basic patch localized to the α1-α3 region (Fig 4E). Collectively, conservation mapping suggests that the domain face formed by α1, α3, α5, and the α6-α7 loop is likely to constitute a functional surface, potentially for protein-protein interactions, mediated by both hydrophobic and electrostatic interactions.

### The SPR2 C-terminal α-solenoid domain is structurally homologous to the C-terminal domains of Ge-1 and the katanin p80 subunit

**Fig 5:**
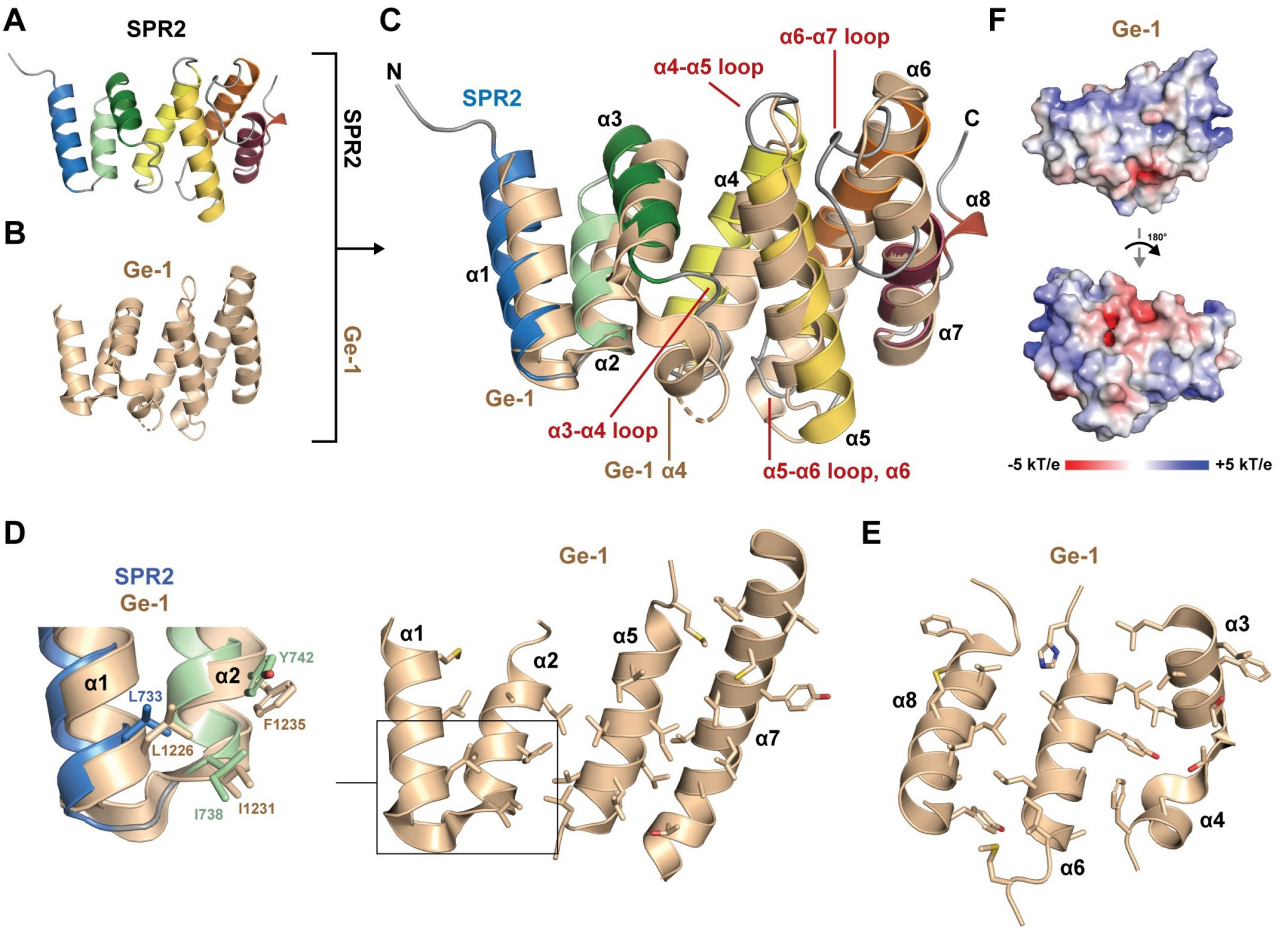
The SPR2 C-terminal domain is structurally similar to the C-terminal domain from the mRNA 5’-decapping factor, Ge-1. (A) Structure of the *A*.*t*. SPR2 C-terminal domain, colored as in Fig 3A, shown in cartoon format. (B) Structure of the *Drosophila melanogaster* Ge-1 C-terminal domain (colored wheat, shown in cartoon format (PDB accession code: 2VXG, Chain A [43]). (C) Structural alignment of the SPR2 C-terminal domain and the *D*.*m*. Ge-1 C-terminal domain from 2VXG [43], oriented as in A and B. Major differences in domain architecture are labeled in red. Labels denote SPR2 secondary structure elements unless otherwise noted. (D-E) Splayed view of the Ge-1 C-terminal domain core, highlighting the residues buried in the core, contributed from helices α1, α2, α5, and α7 (D), and helices α3, α4, α6, and α8 (E), which respectively constitute opposite regions of the domain. The side chains of core residues are shown in stick format. Inset zoom in D compares homologous hydrophobic core residues between SPR2 and Ge-1 in the α1-α2 region, aligned and colored as in C. (F) Electrostatic surface potential mapped on the Ge-1 C-terminal domain structure; top view oriented as in B, bottom view after a 180° rotation about the y-axis.

To determine whether the SPR2 C-terminal domain (residues 717–849) is structurally homologous to other protein structures, we used the Dali server [33] to search the PDB, which identified two highly homologous domain structures: the C-terminal domains from Ge-1 and the katanin p80 subunit. Ge-1 is part of the mRNA 5’ decapping complex, and is involved in localizing the complex to the P-body [41, 42]. The *Drosophila melanogaster* Ge-1 structure (PDB accession code 2VXG, chain A [43]) structurally aligns well with the SPR2 C-terminal domain (Z-score 10.7, 2.6 Å rmsd over 119 Cα atoms, 16% sequence identity) (Fig 5A–5C). The Ge-1 C-terminal domain consists of a core eight α-helices. Ge-1 helices α1-α3 and α5-α8 correspond to SPR2 helices α1-α7 respectively. Ge-1 has a unique α4 helix, positioned perpendicular to α3, that, together with a disordered loop positioned C-terminal to it, bridges the first (α2-α3) and second (α5-α6) helix-turn-helix motifs of Ge-1. Ge-1, like the SPR2 C-terminal domain, has a hydrophobic core that runs along the α-solenoid axis (Fig 5D and 5E), but side chain structural homology to SPR2 is primarily limited to the α1-α2 region (Fig 5D, zoom inset) where Ge-1 residues L1226, I1231, and F1235 are positioned similar to SPR2 residues L733, I738, and Y742 respectively. Additional key structural differences between SPR2 and Ge-1 include (using SPR2 nomenclature) the SPR2 α4-α5 loop, the SPR2 α5-α6 loop (for which the corresponding loop in Ge-1 is flanked by a shorter N-terminal helix, and a longer, and kinked C-terminal helix), and the SPR2 α6-α7 loop (which is extended in SPR2, and in Ge-1 is flanked by a longer C-terminal helix) (Fig 5C). In contrast to SPR2, Ge-1 conservation maps primarily to the opposite face of the domain, including residues on Ge-1 α5 (structurally equivalent to SPR2 α4), and a conserved arginine on Ge-1 α8, which when mutated (R1340E), affects the ability of Ge-1 to localize to P-bodies [43]. The Ge-1 C-terminal domain also has a distinct, net basic electrostatic surface potential (Fig 5F). Overall, the Ge-1 C-terminal domain, while similar to SPR2 in fold, has distinct structural attributes, surface conservation and electrostatics, suggesting that the common fold is involved in distinct, non-overlapping functions for these proteins.

The second hit from the Dali server [33] search of the PDB we performed was the regulatory p80 subunit of the katanin microtubule severing enzyme. Katanin consists of a catalytic p60 subunit and a non-catalytic, regulatory p80 subunit [44]. The p60 subunit has an AAA+ domain that hexamerizes into a lock washer structure that pulls, in an ATP-hydrolysis-dependent manner, on a microtubule lattice β-tubulin tail. Katanin extracts the tubulin subunit from the lattice, leading either to repair (incorporation of GTP-bound tubulin), or lattice destabilization and severing [45–49]. N-terminal to the AAA+ domain is a microtubule-interacting and -trafficking (MIT) domain, which heterodimerizes with the Katanin p80 C-terminal domain [44, 50–52]. As SPR2 and katanin are both involved in reorientation of the plant microtubule array, we compare and contrast the SPR2 and p80 C-terminal domain structures in detail.

**Fig 6:**
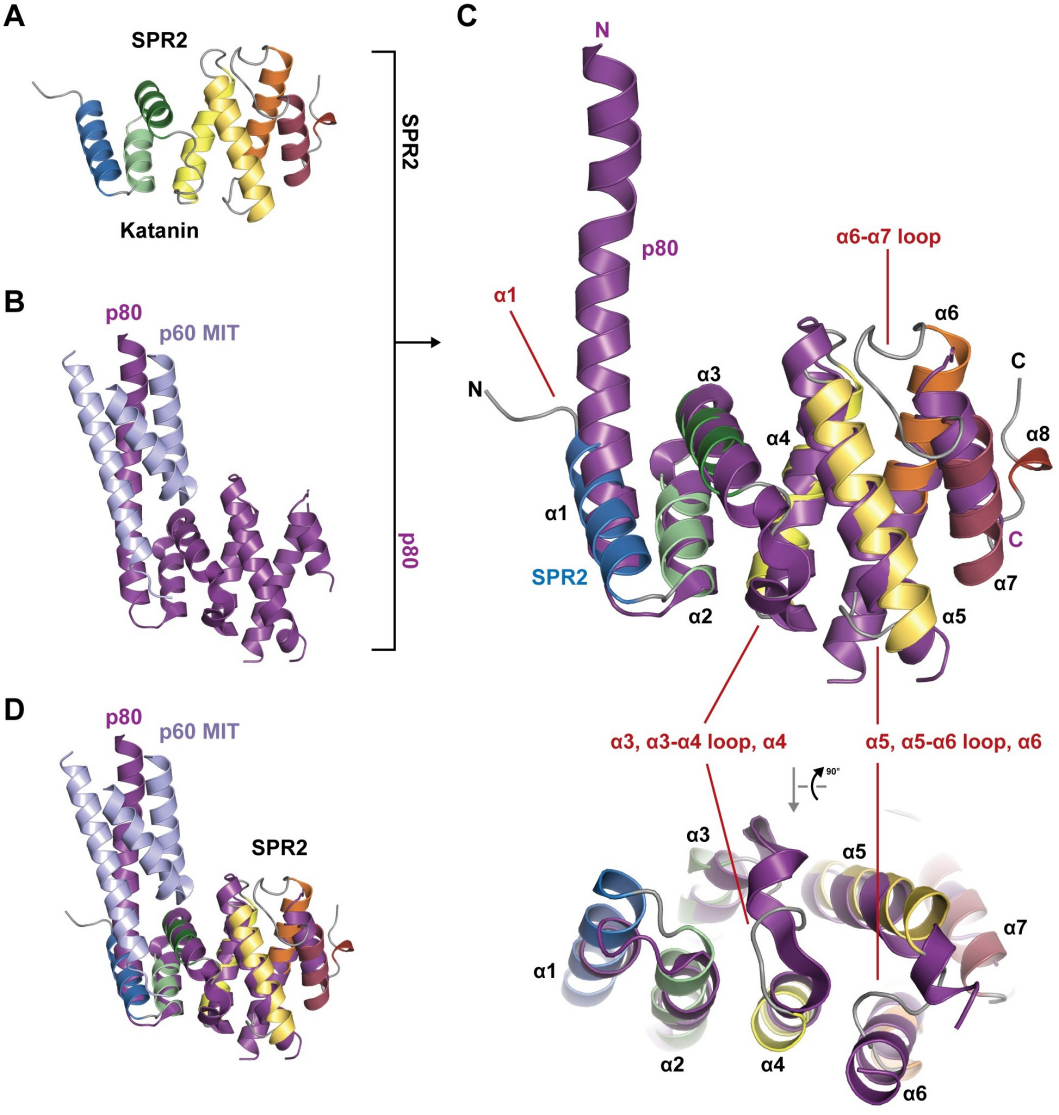
The SPR2 C-terminal domain is structurally similar to the katanin p80 C-terminal domain that heterodimerizes with the katanin p60 MIT domain. (A) Structure of the *A*.*t*. SPR2 C-terminal domain, colored as in Fig 3A, shown in cartoon format. (B) Structure of the mouse katanin p60:p80 heterodimeric complex, involving the p60 MIT domain (colored light blue) and the p80 C-terminal domain (colored purple)(PDB accession code: 5NBT [52]). (C) Structural alignment of the SPR2 C-terminal domain and the katanin p80 C-terminal domain from 5NBT [52], top view oriented as in A and B, bottom view generated by a 90° rotation about the x-axis of the orientation at top, highlighting structural differences in loop conformation between SPR2 and p80. Major differences in domain architecture are labeled in red. Labels denote SPR2 secondary structure elements. (D) Structural alignment of the SPR2 C-terminal domain and the Katanin p60:p80 heterodimer from 5NBT [52], oriented as in A and B.

The SPR2 C-terminal domain aligns well with the p80 C-terminal domain structure, which was determined in complex with the p60 katanin MIT domain (Z-score 9.8, 3.6 Å rmsd over 120 Cα atoms, 13% sequence identity, compared with PDB accession code 5NBT, chain C [52]) (Fig 6A–6C). The p80 C-terminal domain consists of the seven core α-helices that align well with the SPR2 C-terminal domain α-helices. However, we do note the following structural differences. First, p80 katanin α1 has a long N-terminal extension that is involved in binding the p60 MIT domain. Second, the p80 katanin α3-α4 region diverges as follows: the p80 α3 helix is extended relative to SPR2 α3, and the p80 α4 N-terminal region is kinked due to a proline residue in the middle of α4 that contrasts with SPR2’s straight α4 helix. Collectively, these differences position the p80 α3-α4 loop in a conformation distinct from the SPR2 α3-α4 loop (Fig 6C). Third, p80 katanin α6 is shifted relative to SPR2 α6 (along the helical axis), and the loops that flank p80 α6 are disordered (Fig 6C). While the SPR2 α5-α6 loop is ordered, the p80 α5-α6 loop is much longer and includes 15 residues not ordered in the structure. Similarly, the SPR2 α6-α7 loop forms an ordered 11-residue structure that packs against α5, while the 11-residue p80 α6-α7 loop could not be modeled over 10 of the 11 residues.

**Fig 7:**
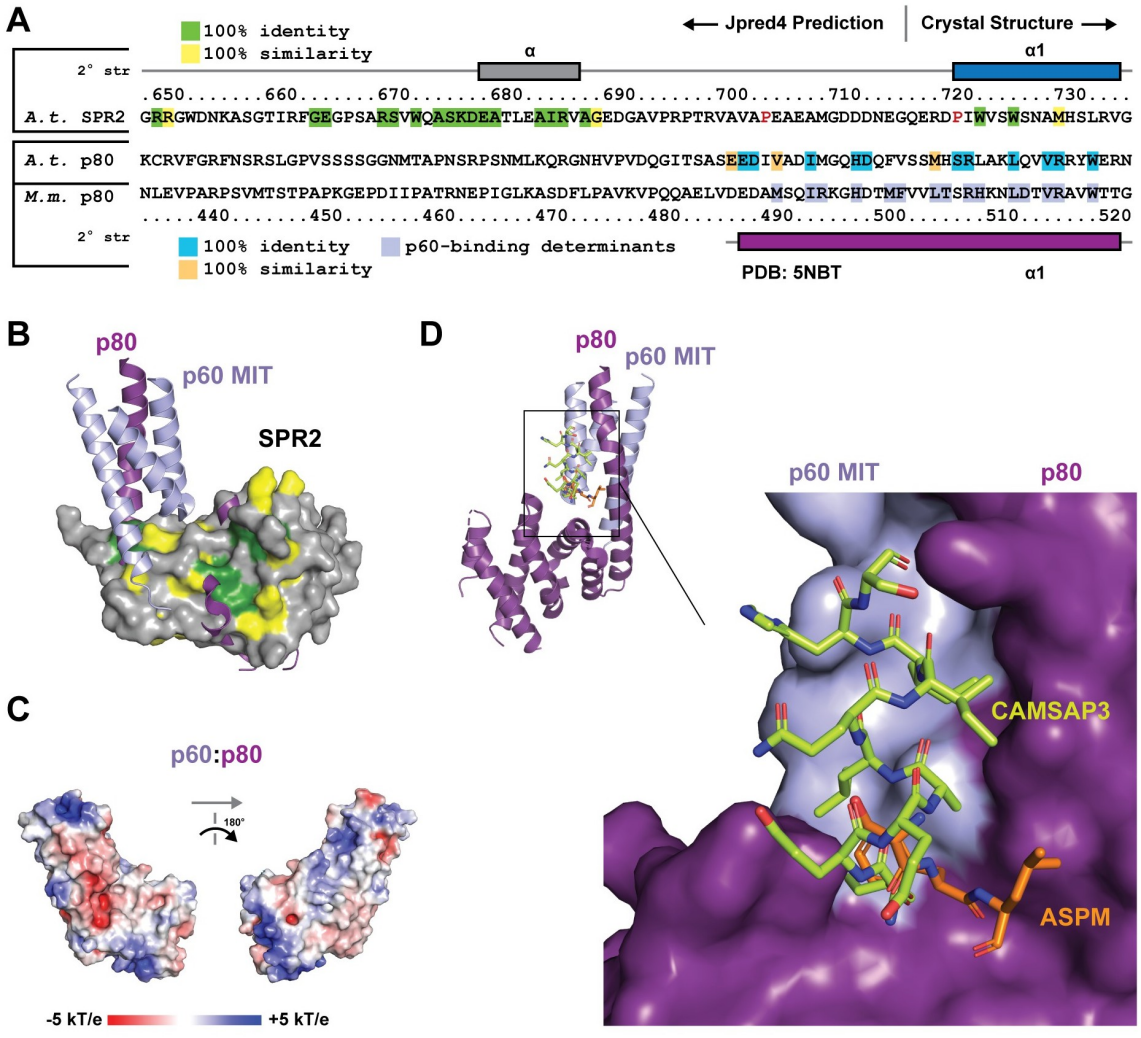
The katanin p80 α1 N-terminal region is distinct from SPR2 α1, and plays a role in binding factors that localize to microtubule minus ends. (A) Sequence alignment of the N-terminal region, including α1, from the *A*.*t*. SPR2 C-terminal domain and the *A*.*t*. and Mus musculus (*M*.*m*.) p80 katanin C-terminal domain. Conservation across SPR2 homologs from Fig 1B is indicated on the SPR2 sequence. *M*.*m*. p80 residues involved in contacts with p60 are highlighted in light blue. Residues conserved between *M*.*m*. p80 and *A*.*t*. p80 over the region modeled in the 5NBT [52] structure are highlighted dark cyan (100% identity) and light orange (100% similarity) on the *A*.*t*. p80 sequence. Residue numbers are for *A*.*t*. SPR2 (above the alignment) and *M*.*m*. p80 (below the alignment). Secondary structure is indicated above for SPR2 based on the crystal structure (residues 717–736), and predicted using Jpred4 (for residues 649–716, which were either not present (degraded) or ordered in the construct crystallized), while the secondary structure for *M*.*m*. p80 is shown below based on the 5NBT structure [52]. Proline residues in the SPR2 sequence that are N-terminal to α1 and within the equivalent span that constitutes α1 in the *M*.*m*. p80 structure are colored red. (B) Structural alignment of the SPR2 C-terminal domain and the katanin p60:p80 heterodimer from 5NBT [52], oriented as in Fig 6D, with SPR2 shown in surface representation with conservation mapped as in Fig 4A. (C) Electrostatic surface potential mapped on the katanin p60:p80 heterodimer structure [52] (left image oriented as in B, right image after a 180° rotation about the y-axis). (D) Structural alignment of the mouse katanin p60:p80 heterodimerization module in complex with the microtubule minus end-binding proteins: Abnormal spindle-like microcephaly-associated protein homolog (ASPM, PDB accession code 5LB7 [51], shown in stick format, colored orange) and CAMSAP3 (PDB accession code 5OW5 [53], shown in stick format, colored chartreuse). The p60 and p80 chains are only shown from the 5LB7 structure for simplicity. Image at upper left depicts p60 and p80 chains in cartoon format with the region boxed in black shown in zoom view (lower right) with p60 and p80 depicted in surface format.

The katanin p60 and p80 subunits form an extensive interaction along the length of p80 α1 [52]. As the interaction with p60 likely stabilized the extended α1 helix, we investigated whether the sequence N-terminal to the SPR2 α1 helix modeled in our structure, might contain homology to the p80 subunit’s α1 p60-binding determinants (Fig 7A), and whether this might suggest an ability of the SPR2 C-terminal domain to directly bind p60. Using the structural alignment as shown in Fig 6C, inclusion of the p60 MIT domain from the 5NBT structure [52] leads to steric clash between the MIT domain and residues on SPR2 α1 and α3 (Figs 6D and 7B). While katanin p60:p80 interactions are primarily hydrophobic, two key hydrophobic residues in p80 α3 correspond with lysine residues in the SPR2 structure, which we anticipate would prohibit p60 and SPR2 from engaging in a similar mode as observed in the katanin p60:p80 heterodimer structure [52]. While many p80 α1 residues involved in p60 binding are conserved between mouse p80 and *A*.*t*. p80, few of these residues are found in SPR2 (Fig 7A). Of note, secondary structure prediction using Jpred4 [28] predicts a disordered region over the SPR2 span equivalent to the p80 α1 N-terminal extension (Fig 7A). This span of SPR2 also includes two proline residues, which would be predicted to compromise formation of a straight helix over the span (Fig 7A). While there is a conserved region 32 residues N-terminal to SPR2 α1, this region has no similarity to p80. Katanin p60 does engage the katanin p80 C-terminal domain over a region that corresponds to a conserved site on the SPR2 C-terminal domain structure involving residues from α1 and α3 (Fig 7B). This suggests that similar regions of the SPR2 and p80 C-terminal domains may be involved in protein-protein interactions. The katanin p60:p80 complex has significant basic electrostatic patches (Fig 7C) that align with the complex’s ability to bind the negatively-charged microtubule exterior [50–52]. This contrasts with the highly acidic electrostatics of the SPR2 C-terminal domain (Fig 4E), but opens the possibility that SPR2 and the katanin p60:p80 complex engage one another using complementary electrostatics. Overall, while the SPR2 and katanin p80 C-terminal domains are structurally similar, they have distinct architectural differences, conservation, and electrostatics. Based on these differences, we do not anticipate that SPR2 engages katanin p60 using a p80-binding mode.

## Conclusion

We experimentally determined the structure of the SPR2 conserved C-terminal domain, revealing a domain fold found in the mRNA de-capping component, Ge-1, and the katanin microtubule severing enzyme regulatory p80 subunit. The SPR2 structure has distinct conformations, conservation, and electrostatics that set it apart from Ge-1 and p80 katanin, suggesting that its function is also distinct. Interestingly, both SPR2 and katanin play central roles in the reorganization of the microtubule array in plants in response to blue light. Katanin is recruited to microtubule crossover sites, where it severs microtubules oriented in the longitudinal array, thereby amplifying the number of microtubules in the longitudinal array [7]. SPR2 recognizes and stabilizes microtubule minus ends, which is critical to prevent depolymerization of the longitudinal array [9–11]. How katanin is recruited to microtubule crossover sites and specifically cleaves the longitudinally-oriented microtubule remains to be fully determined [8], as is the mechanism by which SPR2 specifically binds and stabilizes the microtubule minus end. What role the SPR2 C-terminal domain plays in microtubule minus-end localization remains to be determined, but we note that the domain’s basic patch (Fig 4E) could complement the negatively charged exterior of the microtubule, and is a candidate surface for engagement. Our structural work reveals an interesting evolutionary relation between SPR2 and katanin p80, in that they have a common structural domain. While we do not anticipate binding between SPR2 and katanin p60 in a mode analogous to the katanin p60:p80 complex [51, 52], whether SPR2 and katanin interact remains to be experimentally determined. Interestingly, the mammalian katanin p60:p80 complex uses a common site to bind CAMSAP3 [53] and ASPM [51], two proteins that directly recognize and bind the microtubule minus end (Fig 7D), highlighting the potential evolutionary functional convergence of the katanin p80/SPR2 domain as a determinant at the nexus of microtubule severing and microtubule minus end localization. The SPR2 C-terminal domain structure lays a foundation upon which its role in the regulation of microtubule minus end dynamics and array reorientation can be investigated.
